# Monitoring fever treatment behaviour and equitable access to effective medicines in the context of initiatives to improve ACT access: baseline results and implications for programming in six African countries

**DOI:** 10.1186/1475-2875-10-327

**Published:** 2011-10-31

**Authors:** Megan Littrell, Hellen Gatakaa, Illah Evance, Stephen Poyer, Julius Njogu, Tsione Solomon, Erik Munroe, Steven Chapman, Catherine Goodman, Kara Hanson, Cyprien Zinsou, Louis Akulayi, Jacky Raharinjatovo, Ekundayo Arogundade, Peter Buyungo, Felton Mpasela, Cherifatou Bello Adjibabi, Jean Angbalu Agbango, Benjamin Fanomezana Ramarosandratana, Babajide Coker, Denis Rubahika, Busiku Hamainza, Tanya Shewchuk, Desmond Chavasse, Kathryn A O'Connell

**Affiliations:** 1 Population Services International, Malaria & Child Survival Department, P.O. Box 43640, Nairobi, Kenya, Africa; 2 Population Services International, 1120 19th Street N.W., 20036, Washington D.C., USA; 3 Department of Global Health and Development, London School of Hygiene and Tropical Medicine, 15-17 Tavistock Place, London WC1H 9SH, UK; 4 Associoation Beninoise pour le Marketing Socia/PSI, B.P. 08-0876, Tri Postal, Cotonou, Benin, Africa; 5 Association de Santé Familiale, 232 Avenue Tombalbaye, Immeuble Socir, Kinshasa, Democratic Republic of Congo, Africa; 6 PSI/Madagascar, Immeuble-FIARO, Rue Jules RANAIVO, ESCALIER-D, 2eme Etage, BP 7748, Antananarivo 101, Madagascar, Africa; 7 Society for Family Health, 8 Port Harcourt Crescent, Area 11 Garki Abuja, Nigeria, Africa; 8 PACE, Plot 2 Ibis Vale, P.O. Box 27659, Kololo, Kampala, Uganda, Africa; 9 Society for Family Health, Plot No. 549, Ridgeway, P.O. Box 50770, Lusaka, Zambia, Africa; 10 National Malaria Control Program, Benin, Africa; 11 Malaria National Program, Ministry of Health, Democratic Republic of the Congo, Africa; 12 Ministry of Health, Madagascar, Africa; 13 National Malaria Control Programme, Nigeria, Africa; 14 National Malaria Control Programme, Ministry of Health, Uganda, Africa; 15 National Malaria Control Centre, Ministry of Health, Zambia, Africa

**Keywords:** Malaria, ACT, diagnosis, treatment-seeking behaviour, public sector, private sector

## Abstract

**Background:**

Access to artemisinin-based combination therapy (ACT) remains limited in high malaria-burden countries, and there are concerns that the poorest people are particularly disadvantaged. This paper presents new evidence on household treatment-seeking behaviour in six African countries. These data provide a baseline for monitoring interventions to increase ACT coverage, such as the Affordable Medicines Facility for malaria (AMFm).

**Methods:**

Nationally representative household surveys were conducted in Benin, the Democratic Republic of Congo (DRC), Madagascar, Nigeria, Uganda and Zambia between 2008 and 2010. Caregivers responded to questions about management of recent fevers in children under five. Treatment indicators were tabulated across countries, and differences in case management provided by the public *versus *private sector were examined using chi-square tests. Logistic regression was used to test for association between socioeconomic status and 1) malaria blood testing, and 2) ACT treatment.

**Results:**

Fever treatment with an ACT is low in Benin (10%), the DRC (5%), Madagascar (3%) and Nigeria (5%), but higher in Uganda (21%) and Zambia (21%). The wealthiest children are significantly more likely to receive ACT compared to the poorest children in Benin (OR = 2.68, 95% CI = 1.12-6.42); the DRC (OR = 2.18, 95% CI = 1.12-4.24); Madagascar (OR = 5.37, 95% CI = 1.58-18.24); and Nigeria (OR = 6.59, 95% CI = 2.73-15.89). Most caregivers seek treatment outside of the home, and private sector outlets are commonly the sole external source of treatment (except in Zambia). However, children treated in the public sector are significantly more likely to receive ACT treatment than those treated in the private sector (except in Madagascar). Nonetheless, levels of testing and ACT treatment in the public sector are low. Few caregivers name the national first-line drug as most effective for treating malaria in Madagascar (2%), the DRC (2%), Nigeria (4%) and Benin (10%). Awareness is higher in Zambia (49%) and Uganda (33%).

**Conclusions:**

Levels of effective fever treatment are low and inequitable in many contexts. The private sector is frequently accessed however case management practices are relatively poor in comparison with the public sector. Supporting interventions to inform caregiver demand for ACT and to improve provider behaviour in both the public and private sectors are needed to achieve maximum gains in the context of improved access to effective treatment.

## Background

In malaria endemic settings, children under five are particularly vulnerable to severe disease and death when infected with malaria. Decisions made by children's caregivers at the first signs of potential malaria infection (fever) are critical to ensure child health and survival. Studies of treatment-seeking behaviour illustrate the varied, complex and iterative processes undertaken by caregivers to address fever in children. Most children receive some form of treatment, most often beginning with treatment outside of the formal health care system (e.g. pharmacies, drug shops). In the course of treating a fever episode, multiple treatments are often acquired from a variety of sources [1-3]. In recent years, caregivers have faced new challenges to acquire effective anti-malarial medicines for their children. Cheap and widely available medicines previously relied upon to treat malaria in children, such as chloroquine, are no longer effective. Following guidance from the World Health Organization, by 2009 the vast majority of *Plasmodium falciparum *malaria endemic countries and territories had adopted artemisinin combination therapy (ACT) as national first-line treatments for uncomplicated malaria [4].

Although national policies have changed, use of ACT remains limited in high-burden countries [5]. Results from nationally representative outlet surveys in six sub-Saharan countries (Benin, the Democratic Republic of Congo, Madagascar, Nigeria, Uganda, and Zambia) suggest that when caregivers arrive at outlets searching for fever treatment, they are unlikely to find ACT that they can afford, if they can find ACT at all [6]. While the majority of public and private sector medicine outlets stock anti-malarials, ACT availability is low, particularly in the private sector. In the private sector, quality-assured, first-line ACT is typically six to 21 times as expensive as the most commonly sold/distributed anti-malarial (always a non-artemisinin monotherapy). While availability is generally higher and price generally lower for ACT treatment in the public sector, national sales volume data suggest that the majority of anti-malarial medicines move through the private sector in all countries except Zambia. Market share data also indicate that most anti-malarial medicines sold or distributed in these countries are non-artemisinin monotherapies, such as chloroquine [6].

Financing for malaria control has increased substantially over the last decade. Increased donor and national government spending has facilitated, among other interventions, introduction of rapid diagnostic tests (RDTs) for malaria and ACT treatment. Strategies adopted to improve ACT coverage and use include improved supply of free or heavily subsidized RDTs and pre-packaged ACT in the public and private sectors; public provider training; and behaviour change communication campaigns promoting diagnosis and ACT treatment [7]. One of the global initiatives currently underway is the Affordable Medicines Facility-malaria (AMFm), which aims to expand access to affordable ACT. The AMFm seeks to reduce consumer prices through price negotiations and a buyer co-payment for which both public and private first-line buyers at the country level are eligible. Reduced prices are expected to extend down the anti-malarial supply chain so that when children's caregivers seek fever treatment at a given outlet, they are more likely to find effective medicines that they can afford. Changes in household treatment-seeking behaviour and improved household fever management are expected as access to effective anti-malarials increases. It is further expected that improving access through both public and private sector channels will lead to improvements in fever treatment among children of all socioeconomic backgrounds. The first phase of the AMFm began in 2010; it will operate in eight countries (Cambodia, Ghana, Kenya, Madagascar, Niger, Nigeria, Tanzania and Uganda) for 24 months during which time it is being independently evaluated [8].

This study uses data from household surveys undertaken by *ACTwatch *[9]. *ACTwatch *employs standardized methodologies and questionnaires to monitor national availability, price, volumes and demand for ACT [10]. The data allow comparison of ACT use across countries and provide a baseline for monitoring the success of interventions to increase access such as the AMFm. Three of the six African *ACTwatch *countries are AMFm countries (Madagascar, Nigeria and Uganda), providing the potential to compare countries with and without the subsidy programme over time. This paper presents results from nationally representative household surveys undertaken in Benin, the Democratic Republic of Congo (DRC), Madagascar, Nigeria, Uganda and Zambia between 2008 and 2010, providing data on caregiver behaviour in high malaria burden contexts prior to significant scale-up of affordable ACT. In addition to providing country baselines for monitoring the success of interventions to increase access, these results can also be used to inform the design and implementation of supporting interventions.

## Methods

### Design and sampling

Nationally representative samples were selected using multi-stage cluster sampling, with clusters selected with probability proportional to population size (PPS), irrespective of cluster fever prevalence or estimated number of fevers per cluster. Equal allocation stratification was utilized to allow for strata comparisons, with different strata used depending on the country context. In Madagascar and Zambia, stratification was conducted with urban and rural domains. In the DRC, the sample was stratified across four geographic regions, and in Nigeria, across six geo-political regions. In Uganda, two strata were included for low/moderate and high malaria transmission areas. In Benin, a single domain was used. Within each stratum, 19 sub-districts were selected with PPS from a list of all sub-districts. At the second stage, five enumeration areas (EA) were selected with PPS. The estimated fever prevalence was used to identify the number of households necessary to screen in order to yield the desired number of children with fever. Household sampling frames were not available and therefore, estimated cluster size (obtained from official population listings in each country) was used to identify a sampling interval for systematic random sampling of households within each cluster. A random start household was selected, and the cluster-specific sampling interval was used to guide interviewers in random selection of households (i.e. by walking in a randomly selected direction from the random start household and selecting every n^th ^household according to the sampling interval). Selected households were screened to identify fevers among children under five that occurred in the two weeks preceding the survey. Specifically, a household representative was asked if the household: 1) contained any children under the age of five; and 2) if any of these children had fever during the two weeks preceding the survey. Households with a recently febrile child were included in the study. The total number of households and febrile children included in the study are as follows: Benin, 876 households, 927 children; the DRC, 2, 236 households, 2, 665 children; Madagascar, 1, 961 households, 2, 120 children; Nigeria, 2, 734 households, 3, 274 children; Uganda, 1, 509 households, 1, 752 children; and Zambia, 1, 703 households, 1, 885 children.

### Training and fieldwork

Data collection teams received a five or six-day training focused on administration of the questionnaire and sampling procedures. Data were collected during peak malaria transmission seasons corresponding with the primary rainy seasons in Benin (April-May 2009); the DRC (April-June, 2010); Madagascar (December 2008-January 2009); Nigeria (August-September, 2009); Uganda (March-April, 2009); and Zambia (April-July, 2009). Caregivers of children under five with fever in the two weeks preceding the survey were invited to participate in the study. Respondents were selected based on their responsibilities as primary caregiver for the child with fever (i.e. responsible for daily care of the child including supervision, bathing and feeding). These primary caregivers were typically the child's mother with the exception of orphaned and foster children. An interview lasting approximately one hour was conducted among women that provided informed consent. All questionnaires were reviewed by the team supervisor and at least 20% of all households were re-visited by a supervisor for quality control. Microsoft Access (^© ^Microsoft Corporation, Seattle, WA, USA) was used for double data entry and validation in each country, with the exception of Madagascar, where personal digital assistants programmed using Visual CE software (^© ^Syware, Cambridge, MA, USA) were used for data collection and Microsoft Access for data management. All research activities operated under ethical approval granted by national ethics review boards.

### Materials

Caregivers responded to a series of questions about management of fevers that had occurred among children in their care in the two weeks preceding the survey. Questions documented the type, timing, source and cost of treatments acquired for the child's fever. Caregiver recall and recognition of the type of treatment acquired was aided by the use of a comprehensive anti-malarial field guide with photographs and brand names of common anti-malarials available in public and private sector outlets. A household questionnaire module, modelled after the Demographic and Health Survey (DHS) collected information on housing characteristics and household assets to be used in assessment of relative socioeconomic status [11].

### Measures

Indicators of treatment-seeking behaviour and treatment of fever were constructed for each country from caregiver reports on treatment sources; type of treatments acquired (brand names); timing of treatments given to the child; and whether or not the child received a diagnostic blood test for malaria. Brand names were used to categorize drugs according to generic anti-malarial types (e.g. chloroquine, quinine, artemether-lumefantrine). These were then further classified as artemisinin combination therapy (ACT), artemisinin monotherapy, or non-artemisinin monotherapy. Indicators were calculated using the three classes of anti-malarials above, as well as an overall category for *any anti-malarial*. Consistent with indicators calculated by the DHS and Malaria Indicator Surveys (MIS), anti-malarial (and ACT) treatment received the same or next day after onset of fever was used as a proxy measure for treatment within 24 hours of onset of fever and is considered *prompt treatment*.

Treatment sources were categorized as either public/not for profit or private sector. Public health facilities (PHFs), community health workers (CHWs) and non-profit health facilities were classified as public/not for profit sector, with PHFs constituting the majority of this category. The private sector encompasses outlets with or without qualified health workers (e.g. licensed pharmacies as well as unlicensed drug shops). While treatment-seeking indicators for the fever episode were calculated for each child, anti-malarial type and source were calculated at the drug level as some children received more than one drug to treat one fever episode.

Socioeconomic status was assessed for each household relative to other households using measures of housing, water, sanitation and household asset items modelled after the DHS. A wealth index was constructed from the individual indicators, which were assigned a weight through principal components analysis and standardized in relation to a standard normal distribution. Each child was categorized according to the value of their household's wealth index, and placed in one of five wealth quintiles, ranging from poorest to least poor [12].

### Data analysis

All analyses were performed using individual datasets for each country. Frequencies were tabulated for treatment-seeking and fever treatment indicators. The proportion of children receiving any anti-malarial, an ACT treatment, and a malaria blood test were calculated for each sector. Differences in proportions between sectors were examined using the chi-square test of association. Logistic regression was used to test for an association between household socioeconomic status and 1) ACT treatment, and 2) diagnostic testing. Odds ratios and 95% confidence intervals are reported. Data were weighted to account for difference in the probability of being selected in the different strata. Standard error estimation accounted for clustering at the sub-district cluster and EA levels. Stata 11.0 (^© ^Stata Corp, College Station, TX, USA) was used for all analysis.

## Results

### Sources of fever treatment

Table 1 summarizes treatment-seeking behaviour for children with fever. Caregivers typically respond to fever with some form of treatment, and in some cases treatment is sought from multiple sources. The most common initial response is to visit a private sector outlet in the DRC (51%), Madagascar (53%), Nigeria (46%) and Uganda (42%). By contrast, only 18% of children with fever are initially taken to the private sector in Zambia, where caregivers most commonly initially seek treatment from the public sector (50%). In Benin, caregivers are most likely to initially treat the child at home (44%); this is also a relatively common practice in the DRC (23%), Nigeria (32%), Uganda (38%) and Zambia (25%).

**Table 1:** Treatment-seeking behaviour for children under five with fever in the past two weeks (%)

	Benin	DRC	Madagascar	Nigeria	Uganda	Zambia
	N = 926	N = 2, 661	N = 2, 120	N = 3, 247	N = 1, 695	N = 1, 877
**Initial treatment-seeking behaviour**						
Did not seek treatment	17.4	9.4	9.5	4.8	4.5	8.0
At home	44.2	23.4	15.8	32.2	37.5	24.5
Public sector or non-profit facility, including:	13.5	16.0	21.3	17.6	16.4	49.8
Public/non-profit facility	13.4	16.0	20.1	16.8	14.9	48.6
CHW	0.1	0.0	1.2	0.8	1.5	1.2
Private sector, including:	25.0	51.1	53.4	45.5	41.7	17.7
Private health facility	3.5	10.1	7.1	5.3	28.6	1.9
Pharmacy or drug store	2.8	36.5	27.1	37.1	10.4	5.3
Other private	18.7	4.5	19.3	3.1	2.7	10.5
**At any time during the fever episode**						
Sought treatment outside of the home	50.3	73.3	78.0	72.6	71.7	77.1
**Number of treatment sources (excluding home)**	N = 466	N = 1, 929	N = 1, 660	N = 2, 385	N = 1, 221	N = 1, 437

1 source	91.6	83.8	91.9	88.0	88.3	92.0
2 sources	7.9	15.4	7.6	11.5	11.3	7.6
3 sources	0.4	0.8	0.5	0.6	0.4	0.4
**Sector mix (excluding home)**						
Private, no public	54.9	73.7	68.2	68.7	68.3	21.8
Public, no private	39.9	23.1	28.5	25.7	26.7	72.1
Public & private	5.2	3.3	3.2	5.6	5.0	6.1
**Public sector source, among those who sought public sector treatment**	N = 210	N = 464	N = 518	N = 707	N = 385	N = 1, 094

Public/non-profit facility	98.6	100.0	94.7	95.5	91.1	98.2
CHW	1.4	0.0	5.3	5.0	9.1	2.0
**Private sector source, among those who sought private sector treatment**	N = 280	N = 1, 530	N = 1, 198	N = 1, 797	N = 899	N = 432

Private health facility	22.1	26.1	16.8	13.6	74.1	13.4
Pharmacy or drug store	10.0	70.1	51.6	81.7	21.9	30.6
Other private	71.1	8.6	35.4	8.3	7.0	57.3

Most caregivers eventually seek treatment outside of the home during the course of a fever episode (the DRC, 73%; Madagascar, 78%; Nigeria, 73%; Uganda, 72%; Zambia, 77%). The exception is Benin where caregivers of only half of children with fever seek treatment outside of the home. When seeking treatment outside of the home, caregivers typically visit only one source. The private sector as the sole external source of treatment is far more common than the public sector in the DRC (74% *vs *23%), Madagascar (68% *vs *29%), Nigeria (69% *vs *26%) and Uganda (68% *vs *27%). In Benin, sole treatment outside the home is divided between private (55%) and public (40%) sectors. In Zambia, most caregivers visiting external sources seek treatment in the public sector only (72%) (Table 1).

Among children who receive care at any time during the illness episode, treatment-seeking within the public sector almost always entails a public health facility; CHWs are the source of treatment for fewer than 10% of the children who receive care in the public sector (Benin, 1%; the DRC, 0%; Madagascar, 5%; Nigeria, 5%; Uganda, 9%; Zambia, 2%) (Table 1). Treatment-seeking in the private sector varies by country. Pharmacies and drug shops are the source of treatment for most of the children who receive care in the private sector in the DRC (70%), Madagascar (52%) and Nigeria (82%). In Uganda, private health facilities are the predominant source of treatment for children who receive care in the private sector (74%). Other private outlets, such as kiosks, shops and mobile vendors, are the most common source of treatment for children who receive care in the private sector in Benin (71%) and Zambia (57%) (Table 1).

### Diagnosis and treatment of children with fever

**Figure 1:**
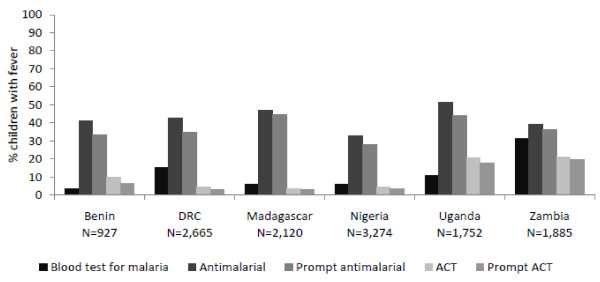
**Percentage of children under five with fever in the past two weeks that received a blood test for malaria, anti-malarial, and ACT treatment**.

Fewer than 10% of children with fever receive a diagnostic blood test for malaria (RDT or microscopy) in Benin (4%), Madagascar (6%) and Nigeria (6%). Diagnosis is slightly more common in the DRC (15%) and Uganda (11%), and in Zambia nearly one-third of children with fever receive a malaria diagnosis (31%) (Figure 1).

Similar levels of anti-malarial treatment for fever are found in Benin (41%), the DRC (43%), Madagascar (47%) and Zambia (39%). In Nigeria, only 33% of children receive anti-malarial treatment and in Uganda, over half receive an anti-malarial (52%). ACT treatment is very low in Benin (10%), the DRC (5%), Madagascar (3%) and Nigeria (5%), but relatively higher in Uganda (21%) and Zambia (21%). When acquired, ACT and other anti-malarial treatments are often provided for children within the same or next day after onset of fever (Figure 1).

### Type and source of anti-malarials acquired for children

**Figure 2:**
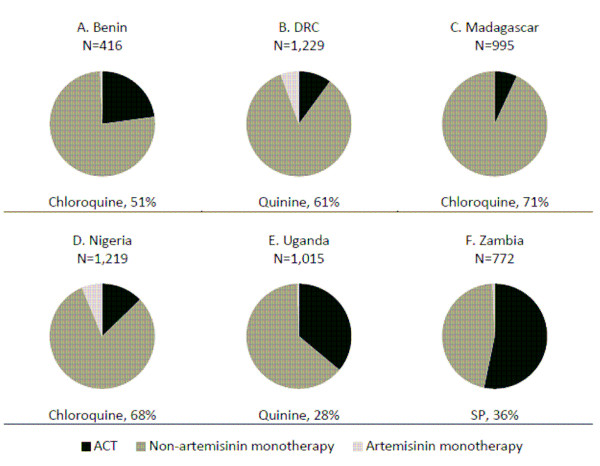
**Type of antimalarials acquired for children under five with fever in the past two weeks (N = number of anti-malarials obtained), and most common non-artemisinin monotherapy (as % of all anti-malarials obtained)**.

Non-artemisinin monotherapies are the most commonly acquired anti-malarial drugs for children under five in Benin (76%), the DRC (84%), Madagascar (93%), Nigeria (81%), and Uganda (63%). The most common non-artemisinin monotherapies are chloroquine (Benin, Madagascar, Nigeria), quinine (the DRC, Uganda), and sulphadoxine/pyrimethamine (SP) (Zambia). While just over half of all anti-malarials acquired for children in Zambia are ACT (53%), ACT makes up a relatively small proportion of all anti-malarial treatments acquired for children in Benin (23%), the DRC (10%), Madagascar (3%), Nigeria (13%) and Uganda (36%). Artemisinin monotherapies make up 1% or less of anti-malarials acquired in all countries except the DRC (6%) and Nigeria (6%) (Figure 2). Most ACT acquired for children are first-line treatments: Benin, 83%; the DRC, 68%; Madagascar, 77%; Nigeria, 88%; Uganda, 100%; and Zambia, 100% (Table 2).

**Table 2:** Percentage of ACT treatments that are the national first-line treatment

	Number of ACT	% first-line treatment
Benin	95	83.2
DRC	131	67.9
Madagascar	83	76.9
Nigeria	171	88.1
Uganda	360	100.0
Zambia	380	99.5

**Figure 3:**
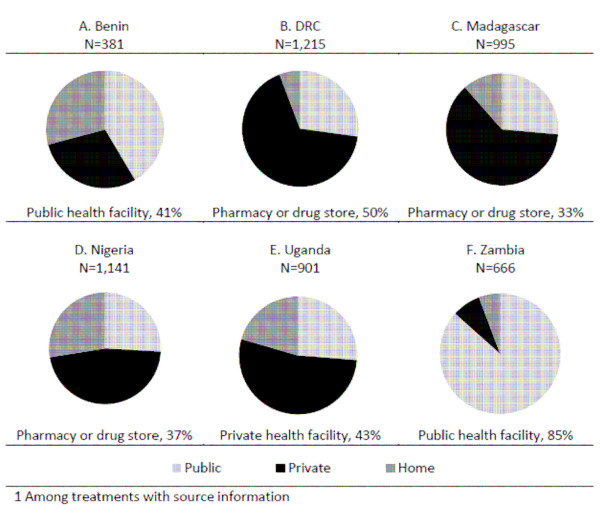
**Source of antimalarials acquired for children under five with fever in the past two weeks (N = number of antimalarials obtained), and most common source (as % of all anti-malarials obtained)^1^**.

The private sector is the source of about half or more of the anti-malarial treatments acquired in the DRC (67%), Madagascar (62%), Nigeria (47%) and Uganda (53%) (Figure 3). Pharmacies and drug stores are the most common private sector source of anti-malarials in the DRC (50% of all treatments acquired), Madagascar (33%), and Nigeria (37%). In Uganda, the most common private sector source is private health facilities (43%). In Zambia, the public sector accounts for 87% of anti-malarial treatments. In Benin, the public sector (41%) is a more common source for anti-malarial drugs than the private sector (29%), and 29% of anti-malarials are reported to be sourced from home. A similar proportion of anti-malarial treatments are sourced from home in Nigeria (28%) and Uganda (20%). "At home" is a less commonly reported source in the DRC (6%), Madagascar (11%) and Zambia (8%) (Figure 3).

### Case management in the public and private sector

Table 3 illustrates differences in diagnosis and treatment of fevers among children who received care either solely in the public sector or solely in the private sector. Across countries, public sector diagnostic testing for malaria and treatment with anti-malarials and in particular, ACT, is low. Fewer than half of children with fevers managed in the public sector receive a blood test for malaria (Benin, 10%; the DRC, 28%; Madagascar, 21%; Nigeria, 14%; Uganda, 21%; and Zambia, 49%). Public sector fever management with an ACT is also low, particularly in Madagascar (7%), Nigeria (10%) and the DRC (11%), and far from ideal in Benin (32%), Uganda (47%) and Zambia (34%).

**Table 3:** Proportion of children under five with fever receiving any anti-malarial, an ACT, and a blood test for malaria, by treatment sector mix

	N	% received anti-malarial	% received ACT	% received blood test
**Benin**				

Public, no private	186	76.3	31.7	9.8
Private, no public	256	43.8	6.6	2.4
χ^2^(1)=		46.82***	47.59***	11.52**
**DRC**				

Public, no private	399	67.2	11.1	27.8
Private, no public	1, 465	45.5	4.0	16.0
χ^2^(1)=		63.70***	31.89***	31.18***
**Madagascar**				

Public, no private	462	52.2	6.7	21.0
Private, no public	1142	55.2	3.2	1.8
χ^2^(1)=		1.25	9.89	177.83***
**Nigeria**				

Public, no private	588	47.6	9.7	14.1
Private, no public	1, 678	35.4	4.0	4.9
χ^2^(1=)		27.97**	27.41***	55.40***
**Uganda**				

Public, no private	322	67.5	46.6	21.3
Private, no public	836	54.3	12.2	11.3
χ^2^(1)=		16.97***	161.42***	18.76*
**Zambia**				

Public, no private	1, 005	57.2	34.0	48.8
Private, no public	343	16.8	5.5	7.3
χ^2^(1)=		157.83***	98.15***	173.01***

* p < 0.05 ** p < 0.01 *** p < 0.001

Although levels of diagnosis and ACT treatment are suboptimal in the public sector, fever management is generally significantly better in the public *versus *private sector. Malaria blood testing is significantly higher among cases managed in the public sector compared to the private sector in all study countries. Fewer than 10% of febrile children managed in the private sector receive diagnostic testing in Benin (2%), Madagascar (2%), Nigeria (5%) and Zambia (7%). Diagnostic testing in the private sector is slightly higher in the DRC (16%) and Uganda (11%) (Table 3).

With the exception of Madagascar, treatment of fever with any anti-malarial and with ACT is significantly higher among children who were treated in the public sector. In Zambia, particularly large differences are seen in both any anti-malarial (public, 57%; private, 17%, χ^2^(1) = 157.8, p < 0.001) and ACT treatment (public, 34%, private, 6%, χ^2^(1) = 98.15, p < 0.001). Differences of a similar scale are seen across sectors in Benin for any anti-malarial (public, 76%; private, 44%; χ^2^(1) = 46.82, p < 0.001) and ACT treatment (public, 32%; private, 7%; χ^2^(1) = 47.59, p < 0.001). In the DRC, Nigeria and Uganda, significant differences in any anti-malarial treatment are observed across sectors, though of a less striking magnitude. However, there are large differences in the proportion of children receiving ACT treatment across sectors in these countries; children seeking care in the public sector are twice as likely to receive ACT treatment as compared to those seeking care in the private sector. In Uganda, the sector difference in ACT treatment is particularly striking: nearly half of children seeking care in the public sector received an ACT (47%) as compared with just 12% managed in the private sector (χ^2^(1) = 161.42, p < 0.001) (Table 3).

### Diagnosis and fever treatment across socioeconomic status

Data from Benin, the DRC, Madagascar, and Nigeria show a pattern of inequity in ACT treatment that disadvantages children living in the poorest households. However, the gradient of disparity according to significant differences in treatment across quintiles is different across these four countries. In Benin and the DRC, it is children living in the wealthiest households that are significantly different from those living in the very poorest (Benin OR = 2.68, 95% CI = 1.12-6.42; the DRC OR = 2.18, 95% CI = 1.12-4.24). Steeper gradients of disparity are observed in Madagascar and Nigeria, where children living in households that fall within the middle of the socioeconomic spectrum are significantly more likely to receive ACT treatment compared to children from the poorest households, and the relatively wealthiest children have particularly higher advantages as compared with the poorest (Madagascar OR = 5.37, 95% CI = 1.58-18.24; Nigeria OR = 6.59, 95% CI = 2.73-15.89) (Table 4).

**Table 4:** Odds of receiving ACT and diagnostic testing by household wealth quintile, among children under five with fever in the past two weeks

	Benin	DRC	Madagascar	Nigeria	Uganda	Zambia
	N = 927	N = 2, 626	N = 2, 120	N = 3, 155	N = 1, 750	N = 1, 886
**Received ACT**						

Lowest	1.00	1.00	1.00	1.00	1.00	1.00
Low	1.15 (0.55-2.39)	1.20 (0.72-2.03)	3.92 (1.35-11.41)*	1.07 (0.40-2.82)	0.62 (0.33-1.17)	0.86 (0.55-1.33)
Middle	1.55 (0.60-3.99)	1.01 (0.46-2.20)	6.75 (1.91-23.85)**	2.97 (1.13-7.83)*	0.58 (0.32-1.04)	0.81 (0.49-1.32)
High	1.30 (0.64-2.65)	1.42 (0.63-3.24)	7.25 (1.55-33.85)*	3.51 (1.44-8.59)**	0.81 (0.38-1.72)	0.47 (0.25-0.87)*
Highest	2.68 (1.12-6.42)*	2.18 (1.12-4.24)*	5.37 (1.58-18.24)**	6.59 (2.73-15.89)***	1.11 (0.56-2.20)	0.62 (0.33-1.14)
**Received blood test**						

Lowest	1.00	1.00	1.00	1.00	1.00	1.00
Low	7.04 (0.75-65.88)	1.61 (0.94-2.76)	1.88 (0.68-5.24)	1.28 (0.63-2.62)	1.76 (0.96-3.24)	1.13 (0.82-1.54)
Middle	4.07 (0.31-53.90)	2.30 (1.41-3.76)**	1.54 (0.62-3.81)	1.19 (0.58-2.43)	1.85 (0.95-3.62)	0.94 (0.64-1.38)
High	7.91 (0.65-96.01)	3.02 (1.80-5.08)***	2.99 (1.16-7.69)*	1.45 (0.80-2.60)	2.00 (1.11-3.59)*	0.76 (0.49-1.19)
Highest	11.87 (1.27-110.58)*	5.92 (3.51-9.98)***	3.16 (1.21-8.22)*	2.58 (1.48-4.50)**	4.53 (2.37-8.64)***	1.06 (0.70-1.60)

* p < 0.05 ** p < 0.01 *** p < 0.001

With the exception of Zambia, children living in the wealthiest households are significantly more likely to receive a malaria blood test compared to children in the poorest households. The odds of receiving a diagnosis among the wealthiest, as compared with the poorest, ranges from 2.58 (95% CI = 1.48-4.50) in Nigeria to 11.87 (95% CI = 1.27-110.58) in Benin. Compared to the poorest children, those living in upper-middle socioeconomic status households are significantly more likely to be tested in the DRC (OR = 3.02, 95% CI = 1.80-5.08), Madagascar (OR = 2.99, 95% CI = 1.16-7.67) and Uganda (OR = 2.00, 95% CI = 1.11-3.59). In the DRC, there is a significant wealth gradient with respect to receiving a diagnostic test (Table 4).

### Caregiver familiarity with effective treatment

While the majority of caregivers in Uganda (57%) and Zambia (81%) can correctly name the first-line anti-malarial treatment for children under five, most caregivers do not have such knowledge in Benin (14%), the DRC (3%), Madagascar (4%) and Nigeria (7%). When asked to name the most effective treatment for malaria in children under five, nearly half of caregivers named the first-line ACT in Zambia (49%), and 35% named the first-line ACT in Uganda. However, very few caregivers named the first-line treatment in Madagascar (2%), the DRC (2%), Nigeria (4%) and Benin (10%) (Table 5).

**Table 5:** Familiarity with and preferences for effective treatment among caregivers of children under five with fever in the past two weeks

	Benin	DRC	Madagascar	Nigeria	Uganda	Zambia
	N = 885	N = 2, 331	N = 1, 970	N = 2, 833	N = 1, 452	N = 1, 727
% Caregivers who name the national first-line drug when asked to list anti-malarials that they have heard of	13.5	3.1	3.5	6.9	57.2	81.1
% Caregivers who name the national first-line drug as the most effective anti-malarial for children under five	9.8	2.0	1.8	4.3	35.1	49.2
	N = 381	N = 1, 186	N = 965	N = 1, 097	N = 898	N = 709

% Children treated with an anti-malarial that received a drug requested by their caregiver	37.5	35.8	51.9	21.3	14.1	7.9
Type of anti-malarial received, among children that received an anti-malarial requested by caregiver^1^	N = 143	N = 450	N = 549	N = 238	N = 127	N = 57

% Non-artemisinin monotherapy	91.6	88.4	95.7	87.8	63.0	75.0
% ACT	10.5	9.2	4.7	7.6	38.1	22.6
% Artemisinin monotherapy	0.0	3.1	n/a	7.6	0.6	2.4

1 Categories are not mutually exclusive as some children received more than one anti-malarial

Beyond familiarity with first-line treatments, another indicator of caregiver knowledge and preferences is the type of treatments requested, if and when caregivers request specific anti-malarial treatments. Fewer than half of caregivers report that they requested by name the anti-malarial drugs that they acquired in Benin (38%), the DRC (36%), Nigeria (21%), Uganda (14%) and Zambia (8%). In Madagascar, 52% of caregivers report requesting the treatment that they received. When caregivers request a specific anti-malarial treatment, they typically ask for non-artemisinin monotherapies. ACT treatment accounts for relatively few of the treatments requested by caregivers across countries (Benin, 11%; the DRC, 9%; Madagascar, 5%; Nigeria, 8%; Uganda, 38%; Zambia, 23%). Artemisinin monotherapies are generally not requested, although 8% of children who receive a requested anti-malarial in Nigeria are treated with artemisinin monotherapy (Table 5).

## Discussion

Children-in whom malarial illness can rapidly progress to severe disease or even death-rarely receive treatment with effective medicines. In Benin, the DRC, Madagascar and Nigeria, ACT treatment is not only uncommon, it is inequitable. Children living in the relatively wealthiest households are more likely to receive effective fever treatment compared to children from the poorest households. In Zambia and Uganda, countries with much higher coverage of treatment with an ACT, still only one in five children with fever received ACT treatment. However, in these countries ACT treatment is not associated with household socioeconomic status. Successful strategies to increase access to ACT in environments of relative political and economic stability are perhaps what set these countries apart.

Zambia was one of the first African countries to change its first-line policy to ACT treatment. Policy change in 2002 was followed rapidly by implementation in 2003 and national scale-up focused on the public health system in 2004. While barriers to accessing public health care in rural areas still exist, the financial barrier that user fees presented was removed in 2006 [13-15]. Furthermore, substantial investment has been made since 2000 by donors, including USAID/President's Malaria Initiative (PMI), GFATM (the Global Fund to Fight AIDS, Tuberculosis and Malaria), the World Bank, and the Bill and Melinda Gates Foundation support through the MACEPA (Malaria Control and Evaluation Partnership in Africa) programme at PATH [7,13].

In Uganda, case management has been strengthened in both the public and private sectors. Government health facility user fees were removed in 2001 followed by an initial increase in utilization of care [16]. The first-line therapy for uncomplicated malaria was changed to the ACT artemether-lumefantrine (AL) in 2004. Pre-packaged AL is provided in public and not-for-profit facilities free of charge. A national home-based management of fever programme was launched in 2002 and modified towards integrated community case management in 2010 [17]. In the private sector, a subsidy pilot launched in 2008 in two districts by the Ministry of Health and the Medicines for Malaria Venture succeeded in improving the availability and reducing the price of ACT [18]. It appears that equitable gains have been achieved in roll-out and scale up of subsidized ACT in the public sector in Zambia and across sectors in Uganda. While a socioeconomic gradient exists in study countries with lower levels of coverage, higher coverage achieved in Zambia and Uganda may contribute to an equitable diffusion to the lower socioeconomic groups that has been observed with high levels of coverage (higher than Zambia and Uganda have yet achieved) [19].

While donor funding for malaria control began to increase in Zambia in 2000 and in Uganda in 2004, substantial donor investment began a bit later in other study countries-in 2005 for the DRC, Nigeria and Madagascar, and in 2007 for Benin [7]. Free public sector provision of ACT treatment was only declared policy in Nigeria in 2009 (the year of the survey), in Madagascar in 2006, and is not yet policy in Benin or the DRC [7]. A private-sector, subsidized ACT programme was launched by Population Services International (PSI) just before the 2008-09 survey in Madagascar. In Nigeria, private-sector, subsidized ACT programmes were first launched by Society for Family Health with funding from the GFATM in 2008 (the year prior to the survey). Public and private sector initiatives supported by increased donor funding in Benin, the DRC, Nigeria and Madagascar appear to have not yet achieved the relative scale and equitable gains observed in Uganda and Zambia. Additional time and investment may be needed in these countries. Political and economic instability may also be playing a role in the DRC, Nigeria and Madagascar.

Further improvement is needed for effective fever treatment among those who seek care in both the private and public sectors across study countries. Implementation of national malaria control policies in the public sector (which in this study is largely comprised of facility-based care as opposed to CHW care), although relatively better than in the private sector, is far from universal. While nearly half of children managed in the public sector in Zambia receive a diagnostic test, testing in the public sector is far lower in all other study countries, ranging from one in ten in Benin to one in four in the DRC. First-line treatment using an ACT has been national policy since 2002 in Zambia; 2004 in Benin, Nigeria and Uganda; 2005 in the DRC; and 2006 in Madagascar. Nevertheless, treatment of fever with an ACT in the public sector is strikingly low in Madagascar (7%), Nigeria (10%) and the DRC (11%), and is far from ideal in Benin (32%), Zambia (34%) and Uganda (47%). Case management practices that do not adhere to national policies have been documented among public health systems in sub-Saharan Africa in a number of studies [20-24]. There is little information on what works to improve provider practices in the public sector [25]. Effective procurement and drug supply management must be in place, including continuous stock of the national first-line drug, as well as absence of ineffective drugs that should not be used to treat malaria. However, supply of effective medicines does not necessarily translate into their use [25-27]. Several studies suggest that training is not adequate to improve provider practices [22,25,26,28,29], even when coupled with job aids [28,29]. Factors including quality of training; integrated rather than vertical training approaches; and quality supervision are thought to be important [22,23,25-27,29,30]. However, there are a number of other factors that may influence provider practices including health worker motivations and perceptions; client factors (e.g. severity of illness, client demands); working environment (e.g. leadership, peers, location, supplies, administration, educational opportunities, competing opportunities); community perceptions, sociocultural traditions and values; and the political and economic environment. Given the myriad of factors with potential to influence behaviour, simply training providers on new guidelines-even when perfectly comprehended, may not translate into provider behaviour change [30]. Interventions far more complex than training and job aid may be necessary. However, these will involve tackling the larger issues that plague public health systems in many developing countries.

Across six countries in sub-Saharan Africa, this study finds that when caregivers search for fever treatment outside of the home they typically visit one outlet [31]. With the exception of Zambia, this outlet is most often part of the private sector. Previous studies have shown that across a range of settings, the private sector is preferred by caregivers seeking treatment given ease of access, reliable drug supply, familiarity with staff, and perceptions around flexible and/or affordable prices [3]. Given that on average, only one drug outlet will be visited, the type of treatments and quality of care available at that outlet are key factors in determining whether or not a child will receive effective treatment. While caregivers often rely on the private sector when seeking treatment, results suggest that fevers are typically more appropriately managed in the public sector. In all countries except Madagascar, children who are treated solely in the public sector are significantly more likely to receive a blood test for malaria and to receive ACT treatment than children who were treated solely in the private sector. Given patterns of caregiver treatment-seeking behaviours that favour the private sector [1], one of the key opportunities to improve fever management in malaria endemic sub-Saharan Africa is to target the private sector. Results from this study show that the types of outlets that dominate private sector treatment-seeking for fever vary across countries. Among those seeking treatment in the private sector, pharmacies and drug shops are more commonly accessed in the DRC, Madagascar and Nigeria. Other outlet types such as shops, kiosks and mobile vendors are more commonly accessed in Benin and Zambia. In Uganda, private health facilities are the most commonly accessed private sector outlet. These results suggest that targeting the private sector to improve fever case management requires context-specific knowledge of the key outlets that are most often visited by children's caregivers for fever treatment.

What exactly is needed to improve case management in the private sector? This study of household treatment-seeking behaviour is limited in the definitive conclusions that can be reached. Results from the *ACTwatch *outlet surveys highlight private sector barriers of availability and price. Across the countries included in this study, ACT availability is significantly lower in the private sector compared to the public sector. Furthermore, when available in the private sector, ACT tends to be substantially more expensive than non-artemisinin monotherapies [6]. Provider practices are also important to consider, particularly as ACT accessibility improves [32-36]. The *ACTwatch *outlet surveys found that, on average, private-sector provider familiarity with the first-line treatment for uncomplicated malaria is significantly lower than knowledge among public providers, and fewer than half of private providers in all six countries can describe the dosing regimen of the first-line treatment for a two-year old child [6]. To leverage the opportunity that the private sector presents for improving fever treatment, both access and provider training are likely to be important. Factors that influence private providers are likely to be different from those that are influential for public sector provider behaviour [30]. Strategies to improve case management will need to include interventions targeted at the unique barriers to appropriate case management among private providers.

Informed demand among children's caregivers may also be a key area for improving fever management in the context of improved private sector access [33-36]. Results from this study illustrate low levels of awareness of the first-line treatment among caregivers, meaning that in the search for fever treatment, they are unlikely to be seeking the first-line drug. Caregivers request anti-malarial medicines by name to varying degrees across countries. However, when they do so, they typically request ineffective medicines, such as chloroquine.

Greater demand for ACT among caregivers could translate to increased market share for these effective treatments, where they are readily available and affordable. Results point to other areas in which informed demand could have a positive impact. Across all countries, treatment is not sought outside of the home for about a quarter of all fevers in children under five. In the case of Benin, half of the children are treated exclusively at home. These results suggest the need for targeted communications around prompt fever treatment with effective medicines that speak to context-specific factors inhibiting existing treatment-seeking behaviour.

'At home' was a frequently cited source for anti-malarial drugs in Benin and Uganda. There is need to understand better what anti-malarial treatment at home entails: are these medicines that have been used to treat a prior illness, in which case there is possibility of incomplete dosing [1], or medicines purchased and stored at home in advance of illness? In addition to creating demand for specific medicines, there may be need for further communication around adherence to full-course treatments.

Communications to raise caregiver awareness and additional provider training may also be needed as diagnostic testing becomes more widely accessible. There will also be need to examine treatment according to diagnostic test results. In this study, blood testing for malaria was generally uncommon and presumptive treatment was still the most informative indicator with respect to fever management. However, WHO policy (2010) recommends parasitological confirmation by microscopy or RDT in all cases of suspected malaria prior to treatment, where diagnostic testing is accessible [37]. In Zambia, the proportion of cases that receive a malaria blood test is slightly higher and approaching 50% among children treated in the public sector. As levels of malaria diagnostic testing rise, presumptive treatment indicators are no longer as informative, given that appropriate treatment of confirmed negative cases must be taken into account. Future studies should focus on describing treatment actions taken according to diagnostic test results. However, bias in caregiver recall of test results is likely to present formidable challenges to this type of measurement, and other methodologies may be needed including routine monitoring systems in the public and private sectors where relevant.

This study aimed to provide key treatment-seeking indicators across countries prior to large-scale efforts to improve access to effective treatment. The nationally representative *ACTwatch *household surveys used standardized methodologies across countries and collected detailed information about treatment-seeking behaviour. Corresponding supply-side data, including availability and price, is available from the *ACTwatch *outlet surveys [6]. These data provide important context to household treatment-seeking behaviour results. Study limitations include reliance on caregiver reports regarding actions taken to treat fever in children. Recall bias was minimized in this study by employing a standard two-week recall period, and using visual aids to prompt identification of treatments acquired for children.

Household survey methodology in and of itself is limiting and often leads to more questions than answers regarding treatment-seeking behaviour. Questions raised in this study include the need for further exploration of the use of anti-malarial medicines that are stored at home. Results show that children treated in the private sector are less likely to receive appropriate diagnostic testing and treatment as compared with children treated in the public sector. Nonetheless, proper case management of fever in the public sector is far from ideal. More information on consumer and provider practices could guide interventions to address both public and private sector deficiencies. Where interventions are developed and implemented, the need for evaluation is great. At present, limited evidence is available on what works for improving provider practices [23,30] and increasing informed demand among caregivers [38]. Additionally, as interventions aiming to increase access to ACT are scaled up, equitable access to treatment monitored through household surveys will be an important indicator of success [39].

## Conclusions

Improved access to ACT is needed in order to achieve equitable gains in effective fever treatment. Treatment-seeking patterns show that the private sector is frequently accessed for fever treatment, and thus could be an important resource for improving access to effective treatment. However, private sector case management practices are relatively poor in comparison with the public sector. Interventions targeting provider practices in both the public and private sectors are needed to support efforts at improving access to ACT. Demand creation targeted at children's caregivers is also an important supporting intervention. As the AMFm and other interventions focused on increased access to ACT scale up, it will be important to monitor the extent to which gains in prompt and effective fever treatment are shared across socioeconomic groups. Additionally, monitoring case management performance in the public and private sectors will be key in evaluating the success of supply-side investments.

## Competing interests

The authors declare that they have no competing interests.

## Authors' contributions

KOC designed the *ACTwatch *household surveys and data collection instruments. ML is responsible for the particular analyses presented in this paper as well as writing the paper. HG, IE, SP, JN, EM and TS made contributions to field work, data cleaning and analyses presented in individual *ACTwatch *household reports. SC, DC, CG and KH made contributions to the study design. CZ, LA, JR, EA, PB and FM supervised data collection and entry. All authors read and approved the final manuscript.
